# The Association between Hand Disinfection Techniques and Their Barriers, as Well as the “Bare below the Elbows” Concept, among Healthcare Professionals—A Study Based on a Polish Population

**DOI:** 10.3390/ijerph191811781

**Published:** 2022-09-18

**Authors:** Emilia Szumska, Przemyslaw Czajkowski, Michal Zablocki, Dorota Rozkiewicz

**Affiliations:** 1 Medilab Sp. z o. o., Niedzwiedzia 60, 15-531 Bialystok, Poland; 2 Clinical Research Centre, Medical University of Bialystok, Jana Kilinskiego 1, 15-089 Bialystok, Poland; 3 Department of Pediatric Infectious Diseases, Medical University of Bialystok, Waszyngtona 17, 15-274 Bialystok, Poland

**Keywords:** disinfection, hand hygiene, antiseptics, infection prevention, bare below the elbows

## Abstract

Hand hygiene is the most effective way to prevent nosocomial infections. Nevertheless, the hands of healthcare professionals are still the primary route of transmission of pathogens responsible for such infections. The aim of this study was to evaluate hand disinfection techniques and investigate the risk factors that may explain the improper hand disinfection techniques among healthcare workers. We selected 7544 hospital workers directly involved in patient care. We recorded data based on the questionnaires, demographic data, and the preparation of hands for disinfection, including risk factors. Correct hand disinfection was verified by *COUCOU BOX*, with a UV camera. Proper hand disinfection was demonstrated among 4879 (64.7%) subjects, while 2665 (35.3%) subjects disinfected their hands incorrectly. In most places of work, nurses properly disinfected their hands more often than the physicians, particularly in general departments (62.1% vs. 69.2%; *p* = 0.0019). We observed that long nails and artificial/polished nails were more often observed in the group of nurses than in the group of physicians (7.3% vs. 4.7%, respectively; *p* = 0.0006 and 19.3% vs. 10.1%; *p* = 0.0000), while an inverse relationship was found in relation to watches (24.0% vs. 12.0%; *p* = 0.0000) and long sleeves (24.4% vs. 8.1%; *p* = 0.0000). Incorrect and less effective hand hygiene among some groups of hospital workers is still present. Therefore, the continuation of education actions concerned with hand hygiene among healthcare workers is needed.

## 1. Introduction

The lack of a proper hand disinfection technique is a leading cause of health care-associated infections, recognized by the World Health Organisation (WHO) as a significant contributor of infectious diseases [1]. Contaminated hands of healthcare workers have been stated to be the leading source of infection transmission in most healthcare facilities [2,3]. According to WHO recommendations, the simplest and most effective and inexpensive way to prevent infection transmission is by washing hands with soap. An alcohol-based hand sanitizer procedure is more effective than a hand washing procedure. Additionally, this procedure reduces more microbes on the hands, is better tolerated by the skin, is faster, and can be performed next to a patient care site, even in areas with no water access [4,5]. Nevertheless, inadequate compliance with hand hygiene procedures is a global problem in developed and developing countries. Nowadays, hand washing and disinfection activities are determined on the basis of 5 points recommended by WHO, namely, before contact with a patient, before aseptic procedures, after contact with body fluids, after contact with a patient, and after contact with the patient’s surroundings [1]. The available literature confirms the increase in the use of alcohol-based hand hygiene products, but the effectiveness of this method is only certain when the disinfection technique is followed [1,6,7].

In the last few years, the phrase “bare below the elbows” (BBE) has become familiar among clinical hospital staff. The recommendations of a BBE policy regarding the dress code of medical personnel who are in patient areas have been accepted by the National Health Service (NHS) and implemented as mandatory in all subordinate healthcare facilities in England [8]. Since nosocomial infections are mainly caused by microbes transmitted by the hands of healthcare professionals, increasing hand hygiene effectiveness through a BBE policy could reduce the number of nosocomial infections. The goal of the BBE policy was to change uniforms in a way that makes it easier for medical personnel to perform hand and wrist disinfection procedures and minimize the risk of microorganism transmission from hands, cuffs, and other clothing. According to the BBE policy, healthcare professionals should wear only short-sleeved clothing, and ties are strictly prohibited. They should take care of the appearance of the nails, which should be short, natural, without varnish or conditioner, etc. In addition, it is forbidden to wear jewelry: watches, bracelets, rings, and wedding rings [8,9]. 

In many European countries, the USA, and Canada, it has been required for years that medical personnel, while working with patients, adhere to a BBE policy. After several years of BBE policy implementation, there is still no unity on these recommendations or their impact on the effectiveness of hand disinfection; there are supporters and opponents of the BBE policy [10,11,12,13,14,15]. Taking the above into consideration, one of the objectives of this study is to assess the impact of a BBE policy on the correctness of hand disinfection procedures and their barriers. Unfortuantelly, evidence from multicenter studies assessing the risk factors of hand hygiene adherence is limited. Szilágyli et al. conducted a study on the correctness of hand disinfection among 5200 employees of the University Hospital in Singapore, with a satisfactory result in 72% of people. Despite the large number of participants in the study, the results of these studies cannot be generalized on a large scale, because they concern only on one hospital [16].

While there is strong evidence that hand hygiene is very important in the prevention of healthcare-associated infections, adherence to the BBE policy and its influence on the effectiveness of hand hygiene is uncertain. The purpose of this study was to evaluate hand disinfection techniques and investigate the risk factors that may explain improper hand disinfection, as well as compliance to the BBE policy among healthcare workers. This study is the first systematic evaluation of hand disinfection techniques among medical and non-medical personnel of Polish origin, not only in hospital units but also in medical clinics, long-term care facilities (LTCFs), and administration offices.

## 2. Materials and Methods

### 2.1. Study Population

The study was carried out in 123 Polish healthcare facilities, diversified in terms of their medical activities, which include medical and non-medical personnel of 12 regions of Poland. All observations were performed among persons working at different parts of the hospital: the medical clinic, long-term care facilities (LTCFs), and administration offices. In the midst of investigated persons, all professional categories were observed: physicians, nurses, and other non-medical personnel (administrative staff, physiotherapists, radiologic technologist, pharmacists, laboratory workers, cleaning staff, and food service employees). In this cohort, we wanted to observe the correctness of hand disinfection, and the influence of risk factors, among medical and non-medical personnel.

### 2.2. Experimental Methods

The study was performed on the volunteers during personnel training dedicated to hand hygiene in two separate study periods: at 2017/2018 and at the end of 2018. The series of personnel training were conducted as a part of the educational campaign “Close the Door to Hospital Infections”, organized by the firm “Medilab” under the auspices of the Polish Scientific Associations and the Scientists of Medical University of Bialystok. The educational campaign organized by Medilab was directed toward medical and non-medical groups for the prevention of nosocomial infections.

Directly after participation in the hand hygiene presentation, volunteers disinfected their hands with 1 dose (3 mL) of the phosphorescent *Aniosgel 85 NPC* (70% Alcohol Denat., Water, Glycerin, Acrylates/C10-30 Alkyl Acrylate Crosspolymer, Bisabolol, Caprylic/Capric Triglycerides PEG-4 Esters, PEG-8 Caprylic/Capric Glycerydes, Aminomethylpropanol, Methylpropanediol). The proper amount (3 mL) of disinfectant was applied by the dispenser on the participants’ hands during the study. The disinfectant was supplemented with a fluorescent substance at different concentrations to guarantee that the total amount of the fluorescent substance on the hands would be the same. We used in our reserch two outside observers/investigators who were well trained and used a validated protocol of the study. Moreover, the observers had no personal relationships with the participants, and no exception of the procedure protocol was allowed. During the procedure, an outside investigator noted anonymously all observations of hand preparation and disinfection. In anonymous questionnaires, during assessments with the UV camera, the sex, job seniority, profession, place of work, (e.g., the kind of medical institution and the hospital ward), the dominant hand, and the proper preparation of hands for disinfection, including risk factors (e.g., a lack of artificial/polished nails, long nails, rings, watches, bracelets, long sleeves, and irritated skin), were recorded. The verification of correct hand disinfection was checked by *COUCOU BOX* (Anios^®^ Laboratories, Lezennes, France), with a UV camera mounted inside the device. The images of hands and wrists were assessed on a computer screen. When 94% of the hand skin area was bright in the UV light camera, correct hand disinfection was indicated. The dorsal and inner sides of the left and right hands were assesed for each volunteer. The palm area was divided into zones, i.e., the fingertip and thumb area, and the lateral, central, and dorsal sides of the hands. All of the zones of both hands accounted for 100% of the hand surface.

### 2.3. Ethical Statement

All of the research methods were carried out in accordance with ethical experimentation standards on humans. The study was conducted according to the guidelines of the Declaration of Helsinki, published in 1975, and revised in 1983. The study protocol was approved by the local Ethics Committee of the Medical University of Bialystok, Poland (R-I-002/180/2017). Before the study inclusion procedure began, written informed consent was acquired from all study participants.

### 2.4. Statistics

Data reported in this article are described as percentages of the number of determinations indicated (n). Groups were compared with Mann–Whitney and one-way analysis of variance (ANOVA) Kruskal–Wallis tests, where appropriate. To compare independent proportions of a normal distribution, a non-parametric chi-square test was used. Determination of the relative probability of inadequate hand hygiene was calculated with the use of an odds ratio (OR) with a 95% confidence interval (95%CI). Statistical analyses were performed by use of Microsoft Excel 2019 and STATA, version 10.0 software (StataCorp, College Station, TX, USA). Differences were considered significant at a *p* value of <0.05.

## 3. Results

### 3.1. Participant Characteristics

The results of 7544 hospital workers, directly involved in patient care, were included in the analysis. Of the staff, 6772 (89.8%) subjects participated in the first study period and 772 (10.3%) particpated in the second study period. We sampled the hands of 6338 women (84.0%) and 1206 men (16%). This included 3801 nurses (72.1%), 1470 physicians (27.9%), and 2273 other personnel (30.1%). Out of all participants, 6896 worked in a hospital (91.4%), 438 worked in a clinic (5.8%), and 210 worked in an LTCF (2.8%). Non-medical staff included the following: cleaning staff: 1016 (44.7%); administrative staff: 405 (17.8%); physiotherapists: 227 (10.0%); food service employees: 225 (9.9%); radiologic technologists: 191 (8.4%); pharmacists: 108 (4.8%); laboratory workers: 101 (4.4%). The general characteristics of the studied population is presented in Table 1.

### 3.2. Hand Hygiene—Assessment of the Correctness of Hand Disinfection Procedures

Statistical analysis showed that the procedure was assessed properly among 4879 (64.7%) subjects, while 2665 (35.3%) subjects disinfected their hands incorrectly. Medical personnel significantly more frequently disinfected their hands properly (3505/5271; 66.5%) compared to non-medical personnel (1374/2273; 60.4%) (*p* = 0.0000). Among medical personnel, nurses disinfected their hands properly significantly more often, compared to physicians (2579/3801; 67.9%, respectively vs. 926/1470; 63.0%) (*p* = 0.0008).

The correctness of hand disinfection was comparable for medical and non-medical personnel: in the hospital (4478/6896; 64.9%), clinic (270/438; 61.6%), and LTCF (131/210; 62.4%) (*p* > 0.05). When employees were divided into medical and non-medical personnel, it was shown that the medical personnel employed in the hospital disinfected their hands properly significantly more often (3237/4831; 67.0%) compared to non-medical personnel (1241/2065; 60.1%) (*p* = 0.0104). No such relationship was found when we compared these two professional groups (medical and non-medical) in the clinic (208/343; 60.6% vs. 62/95; 65.3%—*p* = 0.6920) and LTCF (60/97; 61.9% vs. 71/113; 62.8%—*p* = 0.9441), respectively.

A detailed analysis of the correctness of hand disinfection, among physicians and nurses employed in a hospital, depending on the workplace, was performed (Table 2). Physicians from surgical departments more often disinfected their hands properly (66.6%) compared to physicians employed in general departments (62.1%, *p* = 0.1159), intensive care units (ICUs) (61.5%, *p* = 0.3486), emergency departments (EDs) (56.3%, *p* = 0.2311), and other departments (59.4%, *p* = 0.1567). Moreover, the comparable results of the correctness of hand disinfection were found among nurses employed in individual departments, with the exception of those employed in EDs. Nurses from EDs disinfected their hands properly significantly less often (57.5%) compared to those employed in surgical departments (68.7%) (*p* = 0.0032) and general departments (69.2%) (*p* = 0.0017), ICU (70.5%) (*p* = 0.0052). In most places, nurses disinfected their hands properly more often than physicians, but only in the group of employees working in general departments was the difference statistically significant (62.1% vs. 69.2; *p* = 0.0019), as shown in Table 2.

The correctness of hand disinfection was analyzed among 6896 hospital employees, taking into account the level of the health care referral system (primary, secondary, and tertiary). Statistically significant differences were not found between the staff employed in the primary referral (2579/3968; 65.0%), secondary referral (1139/1726; 66.0%, *p* = 0.4682), or tertiary referral (760/1202; 63.2%, *p* = 0.2618) hospitals. A detailed analysis showed significant differences in the correctness of hand disinfection between physicians and nurses employed in primary and tertiary referral hospitals (respectively 422/678; 62.2% vs. 1419/2071; 68.5%—*p* = 0.0026 and 230/367; 62.7% vs. 373/539; 69.2%—*p* = 0.0408) and between medical and non-medical personnel (1841/2749; 67.0% vs. 738/1219; 60.5%—*p* = 0.0001 and 603/906; 66.6% vs. 157/296; 53%—*p* = 0.0000). In the secondary referral hospitals, the referentiality of such statistically significant relationships was not observed (*p* = 0.8905 and *p* = 0.0646, respectively).

We observed that women more often than men disinfected their hands properly (Table 3), but this difference was not statistically significant (*p* = 0.1152), with the exception of the group of nurses (68.1% vs. 55.8 %; *p* = 0.0227). People who had worked for ≤10 years disinfected their hands with a comparable frequency and correctness to people who had worked longer (*p* = 0.8791). Right-handed people, compared to left-handed people, in all occupational groups disinfected their hands correctly significantly more often (*p* = 0.0000). People who participated in the first study period significantly more often disinfected their hands properly, compared to those who participated in the second study as well (65.4% vs. 58.0%; *p* = 0.0000). The above statement includes nurses (*p* = 0.0458) and non-medical (other) personnel (*p* = 0.0025). In the group of physicians, this difference was not statistically significant (63.8% vs. 55.8%; *p* = 0.0563).

Additionally, the odds ratio (OR) with 95% confidence interval (95%CI) was used to estimate proper hand hygiene among medical and non-medical personnel. Statistical analysis showed that the probability of proper hand disinfection, referring to all tested persons, was significantly increased among nurses (67.8% vs. 61.4%)—OR = 1.32 (95%CI: 1.20–1.46). In addition, the proper preparation of hands for disinfection (71.1% vs. 55.5%), OR = 2.18 (95%CI: 1.98–2.40), and participation in the first study (65.4% vs. 58.1%), OR = 1.37 (95%CI: 1.18–1.59), was also a highly significant predictor of a proper hand disinfection technique. The person’s location of work was a highly significant predictor of a proper hand disinfection technique, observed among medical personnel from surgical departments (67.2% vs. 63.6%), OR = 1.17 (95%CI: 1.06–1.30), especially operating theatres (72.8% vs. 64.2%), OR = 1.49 (95%CI: 1.20–1.86), and ophthalmology departments (70.3% vs. 64.3%), OR = 1.32 (95%CI: 1.08–1.61). Regarding the role of the dominant hand, right-handed persons performed hand disinfection more often than left-handed persons (68.9% vs. 26.7%), OR = 6.03 (95%Cl: 5.08–7.16). Sex (65.1% vs. 62.3%), OR = 1.11 (95%CI: 0.98–1.26), and job seniority (64.6% vs. 64.8%), OR = 0.99 (95%CI: 0.91–1.09), had no influence on the correctness of hand disinfection.

The probability of proper hand disinfection referring to all tested persons was significantly decreased among administration personnel (58.6% vs. 65.5%), OR = 0.74 (95%CI: 0.65–0.86), intensive care unit personnel (57.8% vs. 65.0%), OR = 0.74 (95%Cl: 0.60–0.90), and non-medical personnel representatives (39.2% vs. 66.5%), OR = 0.77 (95%CI: 0.69–0.85).

### 3.3. Risk Factor Determination—Assessment of Hand Preparation and Disinfection Procedure Performance

Analysis of the risk factors showed that only 3932 out of 7544 (52.1%) tested personnel were compliant with the proper hand preparation procedures for disinfection (medical: 2712/5271; non-medical: 1220/2273, *p* = 0.0763). Incorrect preparation of the hands, depending on the risk factor, reduced the chances of correct disinfection. The impact of the risk factors among 7544 personnel on the improper preparation of hands for disinfection is shown in Table 4. Among medical and non-medical (other) personnel, artificial/polished nails (1284/7544; 17.0%), rings (16.5%), bracelets (4.7%), and long nails (6.4%) are noted.

Statistical analysis showed that long and artificial/polished nails were more often observed in the group of nurses than in the group of physicians (7.3% vs. 4.7%, respectively; *p* = 0.0006 and 19.3% vs. 10.1%; *p* = 0.0000), while an inverse relationship was found in relation to wearing watches (24.0% vs. 12.0%; *p* = 0.0000) and long sleeves (24.4% vs. 8.1%; *p* = 0.0000). The presence of rings (*p* = 0.1728), bracelets (*p* = 0.7057), and irritated skin (*p* = 0.1956) was demonstrated with comparable frequency in the group of physicians and nurses (Table 4).

The analysis of job seniority (Table 5) showed that each risk factor was presented more frequently in medical and non-medical personnel with ≤10 years of work experience, compared to personnel with more than 10 years of work experience (*p* < 0.05). In the group of people with ≤10 years of work experience, apart from rings, all risk factors were more common among non-medical personnel. Only in the case of long sleeves was the difference statistically significant: non-medical personnel: 221/349 (63.3%); medical personnel: 367/665 (55.2%) (*p* = 0.0126).

We performed a detailed analysis of the influence on the occurrence of particular irregularities in the proper or improper preparation of hands in the group of physicians and nurses, depending on job seniority. In both professions, all risk factors were significantly more frequent in employees with ≤10 years of work experience. Watches were significantly worn more often by nurses who had worked for less than 10 years in the profession, compared to physicians with a similar amount of employment experience (respectively 307/458; 67% vs. 203/353; 57.5%—*p* = 0.0054). Long nails and artificial/polished nails were significantly more often observed among physicians with ≤10 years of work experience, compared to nurses (long nails: 53/69; 76.8% vs. 160/278; 57.6%—*p* = 0.0033; artificial/painted nails: 109/149; 73.2% vs. 404/734; 55.0%—*p* = 0.0000). In the group of physicians and nurses working for less than 10 years, rings (*p* = 0.8411), bracelets (*p* = 0.9192), irritated skin (*p* = 0.3064). and long coat sleeves (*p* = 0.4744) were observed with comparable frequency.

Further, we performed a statistical analysis to determine the relationship between the presence of risk factors and the amount of training (participation in the study). Generally, the occurrence of such factors as artificial/painted nails (*p* = 0.0290), bracelets (*p* = 0.0053), and irritated skin (*p* = 0.0438) was statistically significantly more frequent in the group of people participating in the second study compared to those in the first study (Table 6). Only long sleeves were found significantly more often in the group of people taking part in the first study (944/6772; 13.9%), compared to those taking part in the second (70/772; 9.1%) (*p* = 0.0002). This relationship concerned both medical personnel (13.0% vs. 9.3%; *p* = 0.0163) and non-medical personnel (16.2% vs. 8.6%; *p* = 0.0016).

An analysis of the impact of the number of risk factors (deviations in the preparation of hands for the procedure) occurring simultaneously in a single person showed that both the presence of one or more (up to six) risk factors had a significant influence on ineffective hand disinfection (Table 7).

The chances of proper hand disinfection increased in the absence of risk factors, OR = 2.18 (95%CI: 1.98–2.40), while it decreased from 16% to 99.97% as the number of risk factors increased: one factor: OR = 0.84 (95%CI: 0.76–0.93); two factors: OR = 0.53 (95%CI: 0.46–0.61); three factors: OR = 0.43 (95%CI: 0.35–0.53); four factors: OR = 0.27 (95%CI: 0.19–0.38); five factors: OR = 0.18 (95%CI: 0.10–0.34); six factors: OR = 0.03 (95%CI: 0.004–0.23).

**Figure 1:**
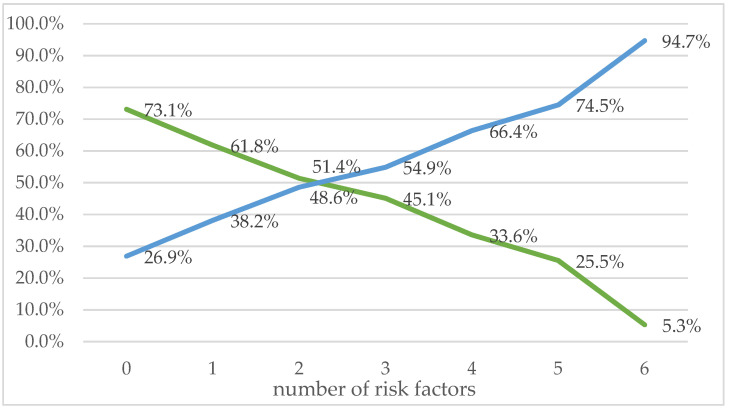
Relationship between the correctness of hand disinfection and the number of risk factors. Blue line—improper hand disinfection; green line—proper hand disinfection.

With the increase in the simultaneous occurrence of risk factors (deviations in the preparation of hands for hygiene procedures), the number of people improperly disinfecting their hands increased: one factor: 802/2100; 38.2%; two factors: 456/938; 48.6%; three factors: 196/357; 54.9%; four factors: 95/143; 66.4%; five factors: 41/55; 74.5%; six factors: 18/19; 94.7% (Figure 1).

## 4. Discussion

To the best of our knowledge, this study represents the first systematic evaluation of hand disinfection techniques among medical and non-medical personnel, not only in hospital units but also in medical clinics, LTCFs, and administration offices. 

Based on the current guidelines, the most frequently recommended disinfectant is alcohol-based hand hygiene products [17]. Disinfection by the use of alcohol prevents the transfer of pathogens between healthcare workers. Several findings have implicated alcohol-based products as effective disinfectants based on (1) a proper amount of alcohol usage, (2) the concentration of alcohol, (3) the type of alcohol, (4) the time during which contact lasts, and (5) the level of hand humidity (wet or not) [18]. For instance, in our study, participants disinfected hands with 3 mL of disinfectant (1 dose); an application of 1 mL is less effective than using 3 mL [19]. Although 3 mL of disinfectant were applied to the volunteers’ hands during the study, not all healthcare workers apply 3 mL to their hands in their clinical practice. This limitation of the study is strictly connected with the clinical setting and the proportion of hands without untreated skin areas.

During the study, the observations of hand disinfection among volunteers was checked by *COUCOU BOX* with a UV camera, Anios^®^. Although the study was conducted with the participation of experienced observers, and with the use of modern equipment, it may be presumed that volunteers were more willing to perform the procedures in the presence of an outside investigator. Taking into account the above considerations, the observers’ experience, and the high requirements for correct hand disinfection procedures, study risk factors were minimized.

Based on the available study data, among all tested personnel, it seems that hospital workers preferably performed hand disinfection. This may be connected to the implementation of regulations and solid experimental and epidemiological data in medical practice. It is more likely that medical staff adopts guidelines if they are based on evidence-based medicine and expert opinions [20]. Moreover, our publication shows that hand disinfection was performed best among surgical department personnel, especially operating theatre staff. This type of information should not be surprising; it is obvious that the practice of modern medical standards is connected to the profession and daily basis activities. Nevertheless, adherence by physicians to hand hygiene guidelines seems to be worse than that of nurses and other healthcare workers [18], as our results have confirmed. 

In view of our results, volunteers who participated in the first study, in comparison to those who also participated in the second study, show proper hand disinfection significantly more often. This shows that training sessions and presentations are associated with an immediate improvement in hand hygiene practices by medical and non-medical personnel.

Many of the variables may potentially interfere with the risk of hand contamination. Previously described risk factors, i.e., wrist watches, bracelets, rings, long sleeves (elements of clothing), long nails, artificial nails, and irritated skin, could affect the correctness of hand disinfection. In 2009, WHO issued appropriate recommendations, which approved short, natural nails, and the absence of rings, wrist watches, and bracelets for effective hand hygiene [21]. In accordance with the recommendations for hand hygiene, it is recommended that long sleeves of protective coats be rolled up before any medical activities [17,21]. A somewhat complex correlation was found with long sleeves among participants in our study. A significant improvement in hand preparation for disinfection was observed, and this observation could be connected to the educational campaign conducted, especially with regard to the presentation of hand hygiene.

As described, WHO recommends natural, unpainted nails for optimal hand hygiene in healthcare facilities. It is particularly emphasized that artificial nails should not be worn during direct contact with a patient [21]. There are relatively minimal reports on painted nails. Nevertheless, after surgical hand disinfection, more bacteria are found on polished nails, as compared to unpolished ones [22]. Therefore, artificial nails (acrylic, gel, or nail tips) have been recognized as a source of infection in several studies [23,24,25,26]. The length of the nails is another hardly defined parameter. Most of the guidance and articles argue that medical personnel should have short nails while caring for a patient. Particularly, in view of recently published papers, long nails increase the total number of germs on the hands of medical personnel [27,28]. Depending on the guidelines, scientists have established a definition of “long nails”: when the length of the nails is 5 [17], 6.3 [21], or 2 mm past one’s fingertips [28]. From a practical point of view, it seems that short nails are those that do not protrude beyond the surface of the skin. For our study, we assume the last sentence as a reference. In our study, it was observed that medical or non-medical staff draw much more attention to the length of nails than to artificial/polished nails, especially during the second study. Based on the obtained results, we can assume that, in the opinion of the respondents, the length of the nails is more crucial in the spread of germs than artificial/polished nails. It would not be an appropriate practice in light of newly published papers [21,22].

Most guidelines do not focus on the presence or absence of bracelets and watches on wrists during hand hygiene procedures. How the presence of bracelets affects the transmission of pathogenic microorganisms and the total amount of pathogens present on the hands of medical personnel has not yet been analyzed. Only the influence of bracelets on the effectiveness of hand disinfection has been determined. It has been proven that the possession of jewelry is significantly associated with poor hand disinfection [7] and makes it difficult to perform a proper hand disinfection procedure [29]. Moreover, it has been confirmed that wearing wrist watches is strictly connected with an increased amount of bacteria on the hands [30], but these data are limited and inconsistent [31]. Furthermore, experts from WHO recommend that medical personnel should remove rings, wedding bands, watches, and bracelets only before surgical preparations of the hands [21]. In many studies, it has been established that wearing rings influences the increased contamination of hands by Gram (+) and Gram (−) bacteria [32,33,34,35]. The above shows that wearing rings during a hand disinfection procedure undoubtedly affects the transmission of bacteria. Unfortunately, the results of the second study did not show that taking off jewelry, such as watches, bracelets, and rings, led to an improvement in the proper preparation of hand hygiene procedures. 

## 5. Conclusions

This study shows that training sessions and presentations are an essential and effective part of the education of medical personnel for improving hand hygiene. From our observations, many issues connected with incorrect and less effective hand hygiene were identified. Our work suggests that not all medical and non-medical personnel are aware of germ spread. Taking the above into consideration, continued education and action concerning hand hygiene among healthcare workers, as well as a further analysis of the conditions of hand disinfection correctness, are needed.

## Figures and Tables

**Table 1:** Study group characteristics.

	Physicians N = 1470	Nurses N = 3801	Others N = 2273	Total N = 7544
Sex				
Female	703 (47.8%)	3724 (98.0%)	1911 (84.1%)	6338 (84.0%)
Male	767 (52.2%)	77 (2.0%)	362 (15.9%)	1206 (16.0%)
Time of Participation in the Study				
First time (1×)	1323 (90.0%)	3431 (90.3%)	2018 (88.8%)	6772 (89.8%)
Second time (2×)	147 (10.0%)	370 (9.7%)	255 (11.2%)	772 (10.3%)
Job Seniority				
≤10 years	911 (62.0%)	2206 (58.0%)	1344 (59.1%)	4461 (59.1%)
>10 years	559 (38.0%)	1595 (42.0%)	929 (40.9%)	3083 (40.9%)
Location of Work				
Hospital	1395 (94.9%)	3436 (90.4%)	2065 (90.8%)	6896 (91.4%)
Clinic	68 (4.6%)	275 (7.2%)	95 (4.2%)	438 (5.8%)
LTCF	7 (0.5%)	90 (2.4%)	113 (5.0%)	210 (2.8%)
Hospital				
Surgical Departments	577 (41.3%)	1264 (36.7%)	367 (17.8%)	2208 (32.0%)
General Departments	589 (42.2%)	1514 (44.1%)	868 (42.0%)	2971 (43.1%)
ED	32 (2.3%)	174 (5.1%)	185 (8.9%)	391 (5.7%)
ICU	91 (6.5%)	261 (7.6%)	47 (2.3%)	399 (5.8%)
Other *	106 (7.7%)	223 (6.5%)	598 (29.0%)	927 (13.4%)
Level of Health Care Referral System				
Primary	678 (48.6%)	2071 (60.3%)	1219 (59.0%)	3968 (57.6%)
Secondary	350 (25.1%)	826 (24.0%)	550 (26.7%)	1726 (25.0%)
Tertiary	367 (26.3%)	539 (15.7%)	296 (14.3%)	1221 (22.0%)

LTCF—long-term care facility; ICU—intensive care unit; ED—emergency department; * other = administration, radiologic technologist, laboratory, or pharmacy.

**Table 2:** Assessment of hand disinfection by physicians and nurses employed in hospital units.

Location of Work	Physicians			Nurses		
	Proper N = 887	Improper N = 508	Total N = 1395	Proper N = 2350	Improper N = 1086	Total N = 3436
Surgical Departments	384 (66.6%)	193 (33.4%)	577 (100%)	868 (68.7%)	396 (31.3%)	1264 (100%)
Operating Theatres	83 (70.9%)	34 (29.1%)	117 (100%)	190 (74.8%)	64 (25.2%)	254 (100%)
Orthopedics	37 (63.8%)	21 (36.2%)	58 (100%)	78 (60.9%)	50 (39.1%)	128 (100%)
Surgery	178 (70.9%)	73 (29.1%)	251 (100%)	385 (64.5%)	212 (35.5%)	597 (100%)
Ophtalmology	86 (57.0%)	65 (43.0%)	151 (100%)	215 (75.4%)	70 (24.6%)	285 (100%)
General Departments	366 (62.1%)	223 (37.9%)	589 (100%)	1048 (69.2%)	466 (30.8%)	1514 (100%)
Neonatology	51 (63.8%)	29 (36.2%)	80 (100%)	111 (66.5%)	56 (33.5%)	167 (100%)
Pediatrics	55 (66.3%)	28 (33.7%)	83 (100%)	101 (70.1%)	43 (29.9%)	144 (100%)
Internal medicine	193 (59.2%)	133 (40.8%)	326 (100%)	685 (71.4%)	275 (28.6%)	960 (100%)
Neurology	43 (66.2%)	22 (33.8%)	65 (100%)	58 (56.3%)	45 (43.7%)	103 (100%)
Rehabilitation	24 (68.6%)	11 (31.4%)	35 (100%)	93 (66.4%)	47 (33.6%)	140 (100%)
ICU	56 (61.5%)	35 (38.5%)	91 (100%)	184 (70.5%)	77 (29.5%)	261 (100%)
ED	18 (56.3%)	14 (43.7%)	32 (100%)	100 (57.5%)	74 (42.5%)	174 (100%)
Other *	63 (59.4%)	43 (40.6%)	106 (100%)	150 (67.3%)	73 (32.7%)	223 (100%)

ICU—intensive care unit; ED—emergency department; * other = administration, radiologic technologist, laboratory, or pharmacy.

**Table 3:** Characteristics of participants properly disinfecting their hands.

	Physicians N = 926	Nurses N = 2579	Others N = 1374	Total N = 4879
Sex				
Female	427/703	2536/3724	1160/1911	4123/6338
	(60.7%)	(68.1%)	(60.7%)	(65.1%)
Male	499/767	43/77	214/362	756/1206
	(65.1%)	(55.8%)	(59.1%)	(62.7%)
Time of participation in the study				
First time	844/1323	2345/3431	1242/2018	4431/6772
	(63.8%)	(68.3%)	(61.5%)	(65.4%)
Second time	82/147	234/370	132/255	448/772
	(55.8%)	(63.2%)	(51.8%)	(58.0%)
Dominant Hand				
Right-handed	875/1269	2498/3481	1310/2061	4683/6811
	(69.0%)	(71.8%)	(63.6%)	(68.8%)
Left-handed	51/201	81/320	64/212	196/733
	(25.4%)	(25.3%)	(30.2%)	(26.7%)
Job seniority				
≤10 years	562/911	1525/2206	795/1344	2882/4461
	(61.7%)	(69.1%)	(59.2%)	(64.6%)
>10 years	364/559	1054/1595	579/929	1997/3083
	(65.1%)	(66.1%)	(62.3%)	(64.8%)
Location of work				
Hospital	887/1395	2350/3436	1241/2065	4478/6896
	(63.6%)	(68.4%)	(60.1%)	(64.9%)
Clinic	34/68	174/275	62/95	270/438
	(50.0%)	(63.3%)	(65.3%)	(61.6%)
LTCF	5/7	55/90	71/113	131/210
		(61.1%)	(62.8%)	(62.4%)
Level of Health Care Referral System				
Primary	422/678	1419/2071	738/1219	2579/3968
	(62.2%)	(68.5%)	(60.5%)	(65.0%)
Secondary	235/350	558/826	346/550	1139/1726
	(67.1%)	(67.6%)	(62.9%)	(66.0%)
Tertiary	230/367	373/539	153/296	756/1221
	(62.7%)	(69.2%)	(51.7%)	(61.9%)

LTCF—long-term care facility.

**Table 4:** Analysis of risk factors among participants who improperly prepare hands for disinfection according to the participant profession.

Risk Factor	Medical Personnel			Other N = 2273	Total N = 7544
	Physicians N = 1470	Nurses N = 3801	Total N = 5271		
long nails	69 (4.7%)	278 (7.3%)	347 (6.6%)	138 (6.1%)	485 (6.4%)
artificial/polished nails	149 (10.1%)	734 (19.3%)	883 (16.8%)	401 (17.6%)	1284 (17.0%)
rings	221 (15.0%)	630 (16.6%)	851 (16.1%)	392 (17.2%)	1243 (16.5%)
watches	353 (24.0%)	458 (12.0%)	811 (15.4%)	292 (12.8%)	1103 (14.6%)
bracelets	63 (4.3%)	172 (4.5%)	235 (4.5%)	121 (5.3%)	356 (4.7%)
irritated skin	98 (6.7%)	293 (7.7%)	391 (7.4%)	132 (5.8%)	523 (6.9%)
long sleeves	358 (24.4%)	307 (8.1%)	665 (12.6%)	349 (15.4%)	1014 (13.4%)

**Table 5:** Analysis of risk factors among participants who improperly prepared their hands for disinfection according to the participant’s job seniority.

Risk Factor	Medical Personnel			Non-Medical Personnel		
	≤10 Years	>10 Years	Total	≤10 Years	>10 Years	Total
long nails	213 (61.4%)	134 (38.6%)	347	89 (64.5%)	49 (35.5%)	138
artificial/polished nails	513 (58.1%)	370 (41.9%)	883	237 (59.1%)	164 (40.9%)	401
rings	540 (63.5%)	311 (36.5%)	851	230 (58.7%)	162 (41.3%)	392
watches	510 (62.9%)	301 (37.1%)	811	198 (67.8%)	94 (32.2%)	292
bracelets	143 (60.9%)	92 (39.1%)	235	79 (65.3%)	42 (34.7%)	121
irritated skin	267 (68.3%)	124 (31.7%)	391	94 (71.2%)	38 (28.8%)	132
long sleeves	367 (55.2%)	298 (44.8%)	665	221 (63.3%)	128 (36.7%)	349

**Table 6:** The frequency of occurrence of the risk factors among the personnel, depending on the time of participation in the study.

Risk Factor	Medical Personnel		Non-Medical Personnel		Total	
	1× * N = 4754	2× N = 517	1× N = 2018	2× N = 255	1× N = 6772	2× N = 772
long nails	322 (6.7%)	25 (4.8%)	124 (6.1%)	14 (5.5%)	446 (6.6%)	39 (5.1%)
	*p* = 0.0916		*p* = 0.6801		*p* = 0.0997	
artificial/polished nails	770 (16.2%)	113 (21.9%)	361 (17.9%)	40 (15.7%)	1131 (16.7%)	153 (19.5%)
	*p* = 0.0011		*p* = 0.3846		*p* = 0.0290	
rings	771 (68.4%)	80 (69.6%)	357 (31.6%)	35 (30.4%)	1128 (16.7%)	115 (14.9%)
	*p* = 0.6624		*p* = 0.1143		*p* = 0.2116	
watches	735 (15.5%)	76 (14.7%)	258 (12.8%)	34 (13.3%)	993 (14.7%)	110 (14.2%)
	*p* = 0.3472		*p* = 0.8052		*p* = 0.7574	
bracelets	201 (4.2%)	34 (6.6%)	103 (5.1%)	18 (7.1%)	304 (4.5%)	52 (6.7%)
	*p* = 0.0140		*p* = 0.1902		*p* = 0.0053	
irritated skin	341 (74.8%)	50 (74.6%)	115 (25.2%)	17 (25.4%)	456 (6.7%)	67 (8.7%)
	*p* = 0.0395		*p* = 0.5335		*p* = 0.0438	
long sleeve	617 (13.0%)	48 (9.3%)	327 (16.2%)	22 (8.6%)	944 (13.9%)	70 (9.1)
	*p* = 0.0163		*p* = 0.0016		*p* = 0.0002	

* Time of participation in the study (1×—first time, 2×—second time).

**Table 7:** Assessment of the correctness of hand disinfection, depending on the number of risk factors (deviations in hand preparation).

The Number of Risk Factors	Proper Hand Disinfection	Improper Hand Disinfection	Total
0	2875 (58.9%)	1057 (39.7%)	3932 (52.1%)
1	1298 (26.6%)	802 (30.1%)	2100 (27.8%)
2	482 (9.9%)	456 (17.1%)	938 (12.4%)
3	161 (3.3%)	196 (7.3%)	357 (4.7%)
4	48 (1.0%)	95 (3.6%)	143 (1.9%)
5	14 (0.3%)	41 (1.5%)	55 (0.8%)
6	1 (0.02%)	18 (0.7%)	19 (0.3%)
Total	4879 (100%)	2665 (100%)	7544 (100%)

## Data Availability

The datasets used and/or analyzed during the current study are available from the corresponding author upon reasonable request.

## References

[1] (2009). WHO Guidelines on Hand Hygiene in Health Care: First Global Patient Safety Challenge Clean Care Is Safer Care.

[2] Boyce J.M., Potter-Bynoe G., Opal S.M., Dziobek L., Medeiros A.A. (1990). A common-source outbreak of Staphylococcus epidermidis infections among patients undergoing cardiac surgery. J. Infect. Dis..

[3] Pittet D., Allegranzi B., Sax H., Dharan S., Pessoa-Silva C.L., Donaldson L., Boyce J.M., Who Global Patient Safety Challenge W.A.f.P.S. (2006). Evidence-based model for hand transmission during patient care and the role of improved practices. Lancet Infect. Dis..

[4] Jumaa P.A. (2005). Hand hygiene: Simple and complex. Int. J. Infect. Dis..

[5] Widmer A.F., Conzelmann M., Tomic M., Frei R., Stranden A.M. (2007). Introducing alcohol-based hand rub for hand hygiene: The critical need for training. Infect. Control Hosp. Epidemiol..

[6] Boyce J.M. (2013). Update on hand hygiene. Am. J. Infect. Control.

[7] Hautemaniere A., Cunat L., Diguio N., Vernier N., Schall C., Daval M.C., Ambrogi V., Tousseul S., Hunter P.R., Hartemann P. (2010). Factors determining poor practice in alcoholic gel hand rub technique in hospital workers. J. Infect. Public Health.

[8] Pratt R.J., Pellowe C.M., Wilson J.A., Loveday H.P., Harper P.J., Jones S.R., McDougall C., Wilcox M.H. (2007). epic2: National evidence-based guidelines for preventing healthcare-associated infections in NHS hospitals in England. J. Hosp. Infect..

[9] Gupta M.K., Lipner S.R. (2020). Hand hygiene in preventing COVID-19 transmission. Cutis.

[10] Dancer S.G., Duerden B.I. (2014). Changes to clinician attire have done more harm than good. J. R. Coll. Physicians Edinb..

[11] Farrington R.M., Rabindran J., Crocker G., Ali R., Pollard N., Dalton H.R. (2010). ‘Bare below the elbows’ and quality of hand washing: A randomised comparison study. J. Hosp. Infect..

[12] Griffin K.J., Scott D.J., Foster N. (2011). Bare below the elbows. Ann. R. Coll. Surg. Engl..

[13] Pace-Asciak P., Bhimrao S.K., Kozak F.K., Westerberg B.D. (2018). Health care professionals’ neckties as a source of transmission of bacteria to patients: A systematic review. CMAJ Open.

[14] Frei A.S. (2015). Bow tie or no tie: A rule to reduce healthcare-acquired infections. J. Community Hosp. Intern. Med. Perspect..

[15] Yildirim I., Ceyhan M., Cengiz A.B., Bagdat A., Barin C., Kutluk T., Gur D. (2008). A prospective comparative study of the relationship between different types of ring and microbial hand colonization among pediatric intensive care unit nurses. Int. J. Nurs. Stud..

[16] Szilagyi L., Haidegger T., Lehotsky A., Nagy M., Csonka E.A., Sun X., Ooi K.L., Fisher D. (2013). A large-scale assessment of hand hygiene quality and the effectiveness of the “WHO 6-steps”. BMC Infect. Dis..

[17] Boyce J.M., Pittet D. (2002). Guideline for Hand Hygiene in Health-Care Settings. Recommendations of the Healthcare Infection Control Practices Advisory Committee and the HIPAC/SHEA/APIC/IDSA Hand Hygiene Task Force. Am. J. Infect. Control.

[18] Nicolay C.R. (2006). Hand hygiene: An evidence-based review for surgeons. Int. J. Surg..

[19] Larson E.L., Eke P.I., Wilder M.P., Laughon B.E. (1987). Quantity of soap as a variable in handwashing. Infect. Control.

[20] Francis R.H., Mudery J.A., Tran P., Howe C., Jacob A. (2016). The Case for Using Evidence-Based Guidelines in Setting Hospital and Public Health Policy. Front. Surg..

[21] Pittet D., Allegranzi B., Boyce J., World Health Organization World Alliance for Patient Safety First Global Patient Safety Challenge Core Group of Experts (2009). The World Health Organization Guidelines on Hand Hygiene in Health Care and their consensus recommendations. Infect. Control Hosp. Epidemiol..

[22] Wynd C.A., Samstag D.E., Lapp A.M. (1994). Bacterial carriage on the fingernails of OR nurses. AORN J..

[23] Foca M., Jakob K., Whittier S., Della Latta P., Factor S., Rubenstein D., Saiman L. (2000). Endemic Pseudomonas aeruginosa infection in a neonatal intensive care unit. N. Engl. J. Med..

[24] Gordin F.M., Schultz M.E., Huber R., Zubairi S., Stock F., Kariyil J. (2007). A cluster of hemodialysis-related bacteremia linked to artificial fingernails. Infect. Control Hosp. Epidemiol..

[25] Gupta A., Della-Latta P., Todd B., San Gabriel P., Haas J., Wu F., Rubenstein D., Saiman L. (2004). Outbreak of extended-spectrum beta-lactamase-producing Klebsiella pneumoniae in a neonatal intensive care unit linked to artificial nails. Infect. Control Hosp. Epidemiol..

[26] Parry M.F., Grant B., Yukna M., Adler-Klein D., McLeod G.X., Taddonio R., Rosenstein C. (2001). Candida osteomyelitis and diskitis after spinal surgery: An outbreak that implicates artificial nail use. Clin. Infect. Dis..

[27] Lin C.M., Wu F.M., Kim H.K., Doyle M.P., Michael B.S., Williams L.K. (2003). A comparison of hand washing techniques to remove Escherichia coli and caliciviruses under natural or artificial fingernails. J. Food Prot..

[28] Rupp M.E., Fitzgerald T., Puumala S., Anderson J.R., Craig R., Iwen P.C., Jourdan D., Keuchel J., Marion N., Peterson D. (2008). Prospective, controlled, cross-over trial of alcohol-based hand gel in critical care units. Infect. Control Hosp. Epidemiol..

[29] Skodova M., Garcia Urra F., Gimeno Benitez A., Jimenez Romano M.R., Gimeno Ortiz A. (2015). Hand hygiene assessment in the workplace using a UV lamp. Am. J. Infect. Control.

[30] Field E.A., McGowan P., Pearce P.K., Martin M.V. (1996). Rings and watches: Should they be removed prior to operative dental procedures?. J. Dent..

[31] Bhusal Y., Laza S., Lane T.W., Schultz K., Hansen C. (2009). Bacterial colonization of wristwatches worn by health care personnel. Am. J. Infect. Control.

[32] Bartlett G.E., Pollard T.C., Bowker K.E., Bannister G.C. (2002). Effect of jewellery on surface bacterial counts of operating theatres. J. Hosp. Infect..

[33] Fagernes M., Lingaas E. (2009). Impact of finger rings on transmission of bacteria during hand contact. Infect. Control Hosp. Epidemiol..

[34] Fagernes M., Lingaas E. (2011). Factors interfering with the microflora on hands: A regression analysis of samples from 465 healthcare workers. J. Adv. Nurs..

[35] Yildirim M., Sahin I., Oksuz S., Sencan I., Kucukbayrak A., Cakir S., Ozaydin C. (2014). Hand carriage of Candida occurs at lesser rates in hospital personnel who use antimicrobial hand disinfectant. Scand. J. Infect. Dis..

